# Iodide‐Mediated Charge Storage in Deep Eutectic Solvent Electrolytes for Nontoxic Printed Supercapacitors

**DOI:** 10.1002/cssc.71194

**Published:** 2026-11-03

**Authors:** Chirag Mevada, Aapo Kattainen, Jari Keskinen, Matti Mäntysalo

**Affiliations:** ^1^ Faculty of Information Technology and Communication Sciences Tampere University Tampere Finland

**Keywords:** activated carbon, charge storage, deep eutectic solvents, potassium iodide, printed supercapacitor, redox‐active electrolytes

## Abstract

Deep eutectic solvents (DESs) are promising sustainable electrolytes for printed energy storage devices owing to their low toxicity, low cost, and tunable physicochemical properties. Herein, a redox‐active DES electrolyte based on choline chloride–urea was developed for nontoxic printed symmetric supercapacitors (SCs) by incorporating potassium iodide (KI) as a charge‐storage enhancer. The influence of KI concentration on electrolyte stability, rheological behavior, and electrochemical performance was systematically investigated. While the addition of 1 wt% water effectively suppressed DES crystallization during storage, KI introduced reversible iodide/triiodide redox reactions that enhanced charge storage beyond conventional electric double‐layer capacitance (EDLC). The DES‐1H_2_O‐2.5KI electrolyte provided a balanced performance, with a specific capacitance of 55 F g^−1^@ 0.1 A g^−1^, an energy density of 24.8 W h kg^−1^, and capacitance retention of 98% after 10,000 cycles. Trasatti analysis revealed a balanced charge storage mechanism with contributions from both EDLC (47%) and pseudocapacitance (53%). In contrast, higher KI concentrations increased electrolyte viscosity and diffusion limitations, resulting in reduced electrochemical efficiency. The results demonstrate that controlled incorporation of iodide redox species provides an effective strategy for improving the performance of DES electrolytes while maintaining electrolyte stability and sustainability. These findings highlight the potential of redox‐active DES electrolytes for sustainable printed SCs.

## Graphical abstract

Iodide‐mediated redox activity in a deep eutectic solvent enhances charge storage in printed supercapacitors. Increasing KI concentration improves capacitance and energy density, while excessive KI increases viscosity and limits ion transport. The results demonstrate the role of electrolyte composition in balancing Faradaic charge storage, ionic transport, and cycling stability.

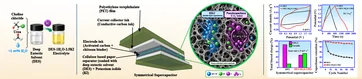

## 1 Introduction

Supercapacitors (SCs) have emerged as important energy storage devices because of their high‐power density, long cycle life, and rapid charge–discharge capability. However, their relatively low energy density compared with that of batteries still limits their broader application in portable electronics, electric vehicles, and grid storage systems [1, 2]. Improving the energy density of SCs has therefore become a major research focus in recent years. One promising approach involves optimizing the electrolyte system since the electrolyte plays a critical role in determining ion transport, electrochemical stability, charge storage mechanisms, and overall device performance. Recent studies have shown that modifications in electrolyte composition can significantly influence capacitance, energy density, and charge‐transfer kinetics in SCs [3].

Deep eutectic solvents (DESs) have recently emerged as attractive alternatives to conventional aqueous and organic electrolytes for electrochemical energy storage systems due to their low cost, low toxicity, and tuneable physicochemical properties [4]. DESs are typically formed by mixing a hydrogen‐bond acceptor and a hydrogen‐bond donor, producing a liquid with a melting point much lower than that of the individual components [5]. Among various DES systems, mixtures based on choline chloride and hydrogen bond donors such as urea or ethylene glycol have attracted considerable attention due to their environmental compatibility, relatively wide electrochemical stability window, and simple preparation process. These characteristics make DES electrolytes promising candidates for sustainable next‐generation electrochemical energy storage devices [4, 6, 7].

A promising strategy to enhance the electrochemical performance of SCs involves introducing redox‐active species into the electrolyte. Unlike conventional inert electrolytes that primarily support electric double‐layer capacitance (EDLC), redox‐active electrolytes participate directly in reversible electrochemical reactions at the electrode–electrolyte interface. These additional Faradaic processes can significantly increase the charge storage capability of the system, leading to improved capacitance and energy density [8, 9]. Recent studies have demonstrated that incorporating suitable redox mediators into the electrolyte provides an effective and relatively simple route for enhancing the electrochemical performance of carbon‐based SCs. Various classes of redox‐active species have been explored for this purpose, including transition‐metal complexes, organic mediators, and halide‐based systems [3]. Among these, iodine‐based redox systems have attracted particular attention because of their fast reaction kinetics, multiple oxidation states, and high reversibility. In such systems, the reversible transformation between iodide ions and triiodide species can provide additional pseudocapacitive charge storage, thereby enhancing the overall electrochemical performance of the device [10]. While many redox‐active electrolytes have been studied in aqueous and organic solvent systems, their behavior in DES environments remains less understood. DES electrolytes possess a unique hydrogen‐bonding network that can strongly influence the electrochemical behavior of dissolved species. For example, Sinclair et al. reported that interactions between DES components and redox‐active molecules can significantly modify redox potentials and reaction reversibility due to hydrogen‐bond stabilization of specific oxidation states. These findings highlight that the compatibility between redox species and the DES environment plays a critical role in determining the electrochemical performance of such electrolyte systems [11]. Among the different iodine‐based systems investigated for SCs, potassium iodide (KI) has received considerable attention as a redox‐active electrolyte component. In our previous work, KI was explored as a redox‐active additive in aqueous‐printed SCs and was found to significantly enhance capacitance through additional iodide‐mediated charge storage [12]. However, the improvement in capacitance was accompanied by higher leakage currents and reduced device lifetime, particularly at elevated operating voltages. These observations highlight a common challenge associated with iodide‐based redox systems, where enhanced energy storage must be balanced against long‐term electrochemical stability. Therefore, developing alternative electrolyte environments that can better stabilize iodide redox reactions while retaining their charge storage benefits remains an important research objective. Recent studies have reported that DES systems can be formed directly from KI and hydrogen‐bond donor molecules such as ethylene glycol, where iodide salts act as key constituents of the solvent structure itself. In these systems, reversible iodine‐iodide transformations contribute significantly to pseudocapacitive charge storage [13].

However, employing iodide salts as the primary component of a DES raises certain concerns regarding electrolyte stability and sustainability. Although DESs are generally considered environmentally benign, the presence of high concentrations of iodide species can lead to the formation of iodine and polyiodide intermediates during electrochemical operation. These species may promote parasitic reactions, increase electrolyte corrosivity, and adversely affect long‐term electrochemical stability. An alternative and potentially more balanced strategy involves introducing iodide species in smaller quantities as redox‐active additives rather than using them as the dominant component of the solvent system. In such an approach, the DES provides a stable and environmentally compatible electrolyte matrix, while a controlled amount of iodide species contributes additional reversible redox reactions that enhance charge storage. This concept allows the beneficial electrochemical behavior associated with iodine redox chemistry to be utilized while minimizing the potential drawbacks associated with iodide‐dominated electrolyte systems. To the best of our knowledge, the use of KI as a redox‐active additive in a choline chloride–urea DES electrolyte for printed symmetric SCs has not been reported previously. Therefore, understanding how controlled KI addition influences ion transport, redox activity, charge storage mechanisms, and electrochemical stability in DES‐based electrolytes remains largely unexplored.

In this context, the present work investigates a choline chloride–urea DES electrolyte containing KI as a redox‐active additive for printed SC applications. By combining the favorable physicochemical properties of the DES with the reversible electrochemical activity of iodide species, the study aims to enhance charge storage while maintaining electrolyte stability. The influence of KI concentration on electrolyte properties, ionic conductivity, electrochemical performance, and charge storage mechanisms is systematically evaluated to identify an optimized electrolyte composition for sustainable printed SCs.

## 2 Experimental Section

### 2.1 Materials

Chitosan obtained from shrimp shells (product 50494), acetic acid, KI, choline chloride, and urea were purchased from Sigma‐Aldrich. Activated carbon powder (YP‐80F) was provided by Kuraray Chemical Co., Japan. A 125 μm‐thick polyethylene terephthalate (PET) film (Melinex ST506, DuPont Teijin Films) was used as the substrate for device fabrication. SIGRAFLEX flexible graphite foil was used as the blocking electrode only for the ionic conductivity measurements. Milli‐Q grade deionized water (Millipore, Merck) was used in all experimental procedures. All chemicals and materials were utilized as received without further purification.

### 2.2 Preparation of Iodide‐Modified DES Electrolytes

DES electrolyte was prepared by mixing choline chloride and urea in a molar ratio of 1:2, following previously reported procedures [4, 14, 15, 16]. The mixture was heated to 80 °C under continuous stirring (VMS C7 Advanced, VWR) until a clear and homogeneous liquid was obtained. After cooling to room temperature, the DES was used as the base electrolyte for the devices. A small amount of DI water (1 wt%) was added to reduce the viscosity of the Reline‐based DES, improve ionic mobility, and suppress crystallization during storage. The water content was kept low to improve the fluidity and physical stability of the DES while maintaining its characteristic properties. Subsequently, KI was incorporated as a redox‐active additive at concentrations of 0.5 wt%, 1 wt%, 2.5 wt%, and 5 wt% relative to the DES electrolyte. Each electrolyte composition was stirred until complete dissolution of KI was achieved, producing homogeneous electrolytes that were used for subsequent electrochemical characterization. The resulting electrolytes were denoted as DES‐1H_2_O‐xKI (*x* = 0.5, 1, 2.5, and 5 wt%) for simplicity in subsequent discussions.

### 2.3 Preparation of Electrode Ink

Electrode ink, referred to hereafter as AC ink, was formulated based on previously reported procedures [17, 18]. A water‐based chitosan binder solution was first prepared by dissolving 1.7 g of chitosan in a mixture containing 67.6 g of deionized water and 0.7 g of acetic acid. The solution was mixed with a magnetic stirrer overnight to ensure complete dispersion and dissolution of the polymer. After preparing the binder solution, the electrode ink was produced by mechanically mixing 6.89 g of the chitosan binder, 3.09 g of activated carbon, and 2 g of deionized water until a homogeneous slurry suitable for printing was obtained.

### 2.4 Fabrication of Symmetrical SCs

The current collectors and electrode layers were deposited onto 125 µm‐thick PET substrates using a blade‐coating technique. A highly conductive carbon ink (LOCTITE EDAG PF 407C E&C) was first applied to form the current collector layer (34 × 31 mm) and subsequently thermally cured at 95 °C for 1 h to ensure good electrical conductivity and adhesion to the substrate. Carbon ink was selected as the current collector material because of its chemical stability in DES electrolytes, flexibility, low cost, and suitability for printed and disposable electronics. After curing the current collector layer, the electrode AC ink was blade‐coated directly onto the conductive carbon (LOCTITE EDAG PF 407C E&C) layer to form the electrode. The coated electrodes had lateral dimensions of 32 × 10 mm and were dried at 60 °C for 30 min to remove residual solvent and stabilize the electrode structure. Finally, two coated substrates were assembled in a face‐to‐face configuration, as illustrated in Figure 1, to form the device. Dry thicknesses of ≈20 µm were obtained for both the current collectors and the electrodes. The devices were assembled by positioning a cellulose‐based paper separator (Dreamweaver Titanium AR40 cellulose paper) impregnated with the DES electrolyte between the two electrodes, followed by sealing the structure using an adhesive transfer tape (3M 468 MP). The total mass of the active electrode material was determined by measuring the weight of the substrate before and after deposition of the electrode layer and calculating the corresponding mass difference. The total electrode active material loading for the symmetric SC device was ≈30 mg.

**FIGURE 1:**
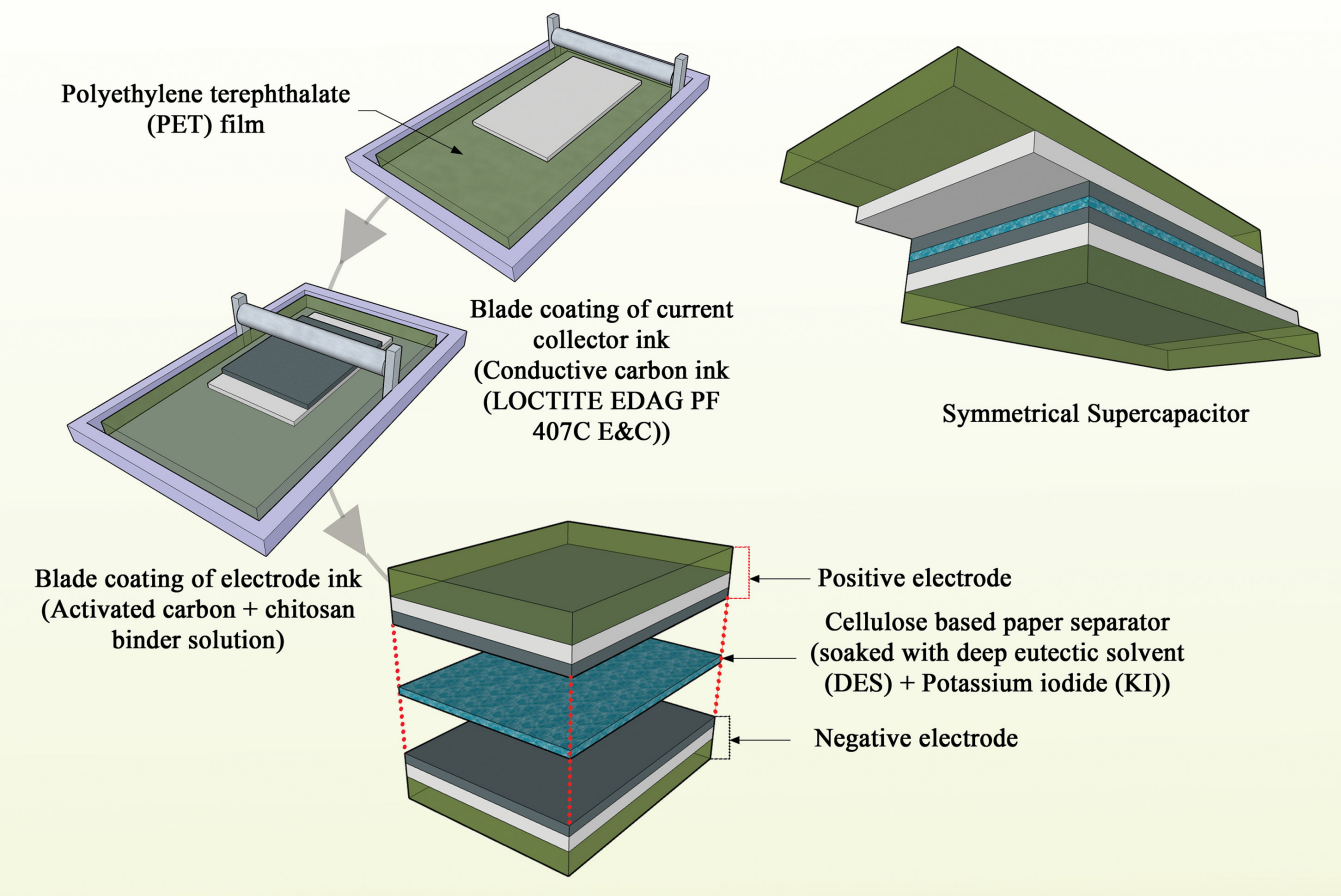
Schematic representation of the SC fabrication process.

### 2.5 Electrochemical Characterization

The electrochemical performance of the SC devices was evaluated using galvanostatic charge–discharge (GCD), cyclic voltammetry (CV), and electrochemical impedance spectroscopy (EIS). GCD and CV measurements were performed using a Maccor 4300 electrochemical workstation. The charge–discharge tests were conducted at constant currents of 1 mA (0.05 A g^−1^), 3 mA (0.1 A g^−1^), and 10 mA (0.3 A g^−1^). CV measurements were carried out at scan rates of 5, 10, 50, and 100 mV s^−1^. CV measurements were carried out at low scan rates ranging from 1 to 30 mV s^−1^ to perform the Trasatti analysis and evaluate the EDLCs and Faradaic contributions. The EIS measurements were performed over a frequency range of 10 mHz to 1 MHz with an AC perturbation amplitude of 5 mV. Both CV and EIS measurements were conducted using a Zahner Zennium potentiostat. Cycling stability measurements were performed at a constant current density of 1 A g^−1^ using GCD on a Maccor 4300 electrochemical workstation.

Capacitance and equivalent series resistance (ESR) values were determined from GCD measurements in accordance with the IEC 62391‐1 standard [18]. The specific capacitance was calculated using the Equations (1) and (2) from CV and GCD, where the SC was discharged at a constant current of 1 mA, and the discharge time (Δ*t*) was measured between 80% and 40% of the maximum operating voltage. The ESR was evaluated from the initial voltage drop (IR‐drop) observed at the start of a 10 mA constant current discharge using Equation (3). Energy density (W h kg^−1^) and power density (kW kg^−1^) of device were calculated from the GCD data using Equations (4) and (5) [19].

(1) $$C_{\mathrm{cv}}\text{ =}\;\frac{\int i(V)\;\text{d}V}{2\text{ν}m\text{Δ}\text{V}}$$

(2) $$C_{\mathrm{GCD}}\text{ = }\frac{I\text{Δ}t}{m\text{Δ}V}$$

(3) $$\mathrm{ESR}\text{ =}\;\frac{\text{Δ}V}{I}$$

(4) $$\text{Energy density =}\;\frac{C_{\mathrm{GCD}}\;\times \;\text{Δ}V^{2}}{2\times 3600}$$

(5) $$\text{Power density =}\;\frac{V^{2}}{4\;\times R_{\mathrm{ES}}\times m}$$

where ∫i(V) *d*V represents the integrated current over the potential window, corresponding to the area under the current‐potential (CV) curve; i(V) represents the current as a function of the applied potential V, ν denotes the scan rate in mV s^−1^, m is the total mass of the electrode material in g, and Δ*V* is the potential range in V, and *R*_ES_ is the equivalent series resistance. The leakage current was assessed by maintaining the device at its maximum operating voltage for 1 h, after which the steady‐state current was recorded at the end of the holding period. The maximum operating voltage was limited to 1.8 V, which is corresponding to the electrochemical stability window of the DES electrolyte.

## 3 Results and Discussion

To evaluate the influence of electrolyte composition on the electrochemical performance of the SCs, a systematic investigation was performed by modifying the DES with small amounts of water and KI. Initially, the electrochemical behavior of the pristine DES electrolyte was evaluated to establish a baseline for comparison. Devices using the pristine DES exhibited typical EDLC behavior, delivering a specific capacitance of ≈37 F g^−1^ and an energy density of around 17 W h kg^−1^. These results indicate that charge storage in the pristine DES electrolyte is primarily governed by electrostatic ion adsorption at the electrode–electrolyte interface. During storage under ambient laboratory conditions (~23 °C and relative humidity of 17.6%–25.8%), the pristine DES gradually formed crystalline or solid‐like phases within ≈3 days. Such behavior is commonly associated with the strong hydrogen‐bond network present in reline‐type DES systems. In the choline chloride–urea mixture, chloride ions act as hydrogen‐bond acceptors, while urea molecules act as hydrogen‐bond donors, forming an extended supramolecular network that can promote structural ordering and crystallization during storage. To improve the physical stability of the electrolyte, 1 wt% DI water was intentionally introduced into the DES system. The addition of a small amount of water helps disrupt the extensive hydrogen‐bond network, thereby reducing intermolecular ordering and preventing crystallization of the electrolyte. The resulting improvement in electrolyte fluidity and homogeneity also facilitates stable electrochemical operation within the investigated voltage window of 1.8 V. Digital images recorded at regular intervals (Figure 2a,b) confirm that the DES containing 1 wt% H_2_O remains in a stable liquid form, whereas the water‐free DES gradually solidifies. Similar stabilization effects of water in Reline‐type DES (choline chloride–urea) electrolytes have been reported in previous studies, where water reduces viscosity and weakens hydrogen‐bond interactions [4].

**FIGURE 2:**
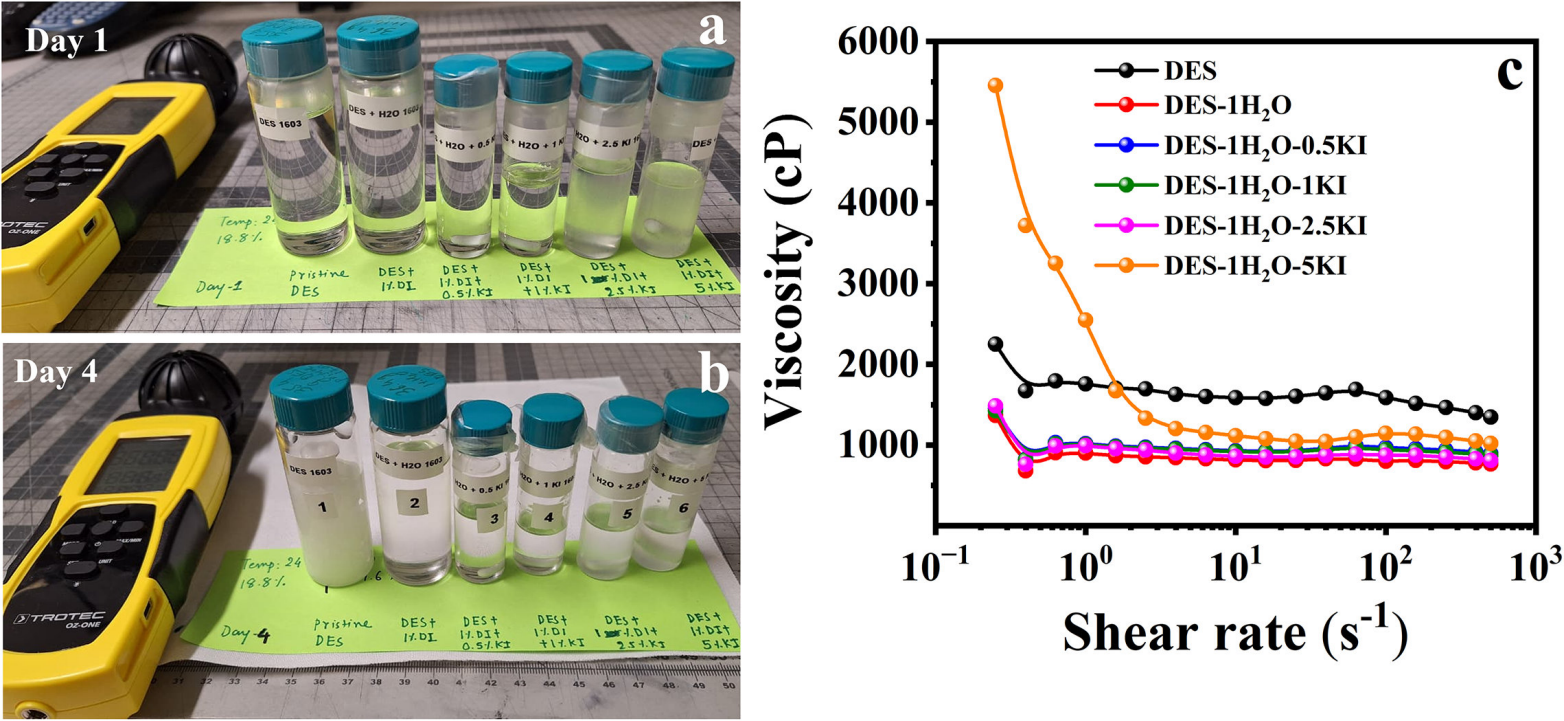
Photographs of the electrolytes after (a) day 1, (b) day 4 of storage showing the stability and crystallization behavior at different KI contents and (c) viscosity as a function of shear rate for DES, DES‐1H_2_O, DES‐1H_2_O‐0.5KI, DES‐1H_2_O‐1KI, DES‐1H_2_O‐2.5KI, and DES‐1H_2_O‐5KI electrolytes, demonstrating the effect of KI concentration on rheological properties.

Despite improving electrolyte stability, the addition of 1 wt% H_2_O had a negligible effect on charge storage, as the specific capacitance remained almost unchanged from 37.8 ± 1.5 F g^−1^ for pristine DES to 37.9 ± 1.3 F g^−1^ for DES‐1H_2_O (Table 1). Thus, the main benefit of 1 wt% H_2_O was improved electrolyte stability and fluidity rather than increased capacitance. This observation is consistent with previous reports by Azmi et al., who demonstrated that the addition of small amounts of water mainly improves ion transport in highly open electrode structures such as 3D graphene, whereas the effect is less pronounced for microporous activated carbon electrodes [4]. After stabilizing the DES electrolyte with 1 wt% water, KI was introduced as a redox‐active additive in concentrations ranging from 0.5 to 5 wt% to enhance the charge storage capability. The influence of KI concentration on the electrochemical performance is summarized in Table 1. The CV curves recorded at 5 mV s^−1^ (Figure 4a) show that the pristine DES and DES‐1H_2_O electrolytes exhibit nearly rectangular profiles, characteristic of ideal EDLC behavior. Upon addition of KI, the CV curves gradually deviate from the rectangular shape and display increased current response, indicating the emergence of additional Faradaic contributions associated with the reversible iodide/triiodide redox couple. As the KI concentration increases, the current density in the CV curves also increases, reflecting improved charge storage capability. Among the tested compositions, the electrolyte containing 2.5 wt% KI exhibits the most favorable electrochemical response, showing a significantly enlarged CV area while maintaining a relatively symmetric profile. This indicates a balanced contribution from both EDLC and redox pseudocapacitance. Further insight into the electrochemical behavior was obtained from GCD measurements, as shown in Figure 4b. Devices using the pristine DES and DES‐1H_2_O electrolytes exhibit nearly triangular charge–discharge profiles typical of EDLC systems. In contrast, the addition of KI results in longer discharge times and slight curvature in the voltage profiles, indicating the presence of additional Faradaic processes. The device containing 2.5 wt% KI demonstrates the longest discharge time, corresponding to the highest specific capacitance among the investigated systems. These electrochemical trends are further supported by rheological measurements (Figure 2c), which show that the addition of 1 wt% water significantly reduces the viscosity of the pristine DES (≈2200cP at low shear rates) by partially disrupting the hydrogen‐bond network between choline chloride and urea, thereby improving electrolyte fluidity and ion transport. KI addition up to 2.5 wt% causes only minor changes in viscosity, suggesting that ion mobility remains sufficiently high for efficient charge transport within the porous activated carbon structure. In contrast, the DES‐1H_2_O‐5KI electrolyte exhibits distinctly different flow behavior, particularly at low shear rates, where its substantially higher viscosity indicates reduced fluidity. This behavior is consistent with stronger interactions between dissolved ions and a more structured electrolyte environment. As a result, although more redox‐active iodide species are available, their effective utilization becomes limited because ion movement through the porous electrode becomes slower. Consequently, the 5 wt% KI electrolyte shows only a marginal increase in current response in CV measurements without a proportional improvement in overall electrochemical performance. Furthermore, at higher KI concentrations (5 wt%), partial crystallization was observed after ≈1 week of storage, with small crystalline deposits appearing at the bottom of the electrolyte (Figure 2b). This behavior likely reflects the limited solubility of KI in the DES matrix and stronger ion–ion interactions at elevated salt concentrations, which promote gradual salt aggregation and precipitation during storage. These observations highlight the importance of electrolyte optimization, where moderate KI concentrations (2.5 wt%) provide a balance between electrolyte stability, ionic transport, and redox activity, resulting in improved electrochemical performance without long‐term phase instability. To further investigate the influence of KI addition on ion transport, the ionic conductivity of the pristine DES, DES‐1H_2_O‐2.5KI, and DES‐1H_2_O‐5KI electrolytes was evaluated using electrochemical impedance spectroscopy. The measurements were performed using a Swagelok‐type conductivity cell equipped with two SIGRAFLEX flexible graphite foil blocking electrodes, separated by an electrolyte‐soaked cellulose separator by an electrolyte‐soaked cellulose separator (Figure 3a,b). The ionic conductivity was calculated from the bulk resistance obtained from the high‐frequency intercept of the Nyquist plot according to Equation (6) [7, 20].

**TABLE 1:** Electrochemical performance parameters of DES‐based electrolytes with KI redox additives.

Electrolyte composition	**Specific capacitance, ‍F ‍g** ^**−1**^	ESR, Ω	**Leakage to capacitance, ‍µA ‍F** ^**−1**^	**Energy ‍density, W h kg** ^**−1**^	**Power‍‍density, kW kg** ^**−1**^	Cyclic ‍stability after (10,000)
DES	37.8 ± 1.5	20.5 ± 2.0	90.8	17.0 ± 0.4	1.7 ± 0.1	84%
DES‐1H_2_O	37.9 ± 1.3	21.6 ± 2.4	84.1	17.1 ± 0.8	1.5 ± 0.2	82%
DES‐‍1H_2_O‐‍0.5KI	46.3 ± 2.1	20.1 ± 2.3	175	20.8 ± 0.7	1.7 ± 0.15	96%
DES‐‍1H_2_O‐‍1KI	47.6 ± 2.4	24.0 ± 3.2	350	21.4 ± 0.8	1.6 ± 0.1	98%
DES‐‍1H_2_O‐‍2.5KI	55.0 ± 3.0	21.4 ± 2.1	230	24.8 ± 0.9	1.2 ± 0.1	98%
DES‐‍1H_2_O‐‍5KI	74.9 ± 4.2	24.7 ± 3.1	315	33.7 ± 1.2	1.0 ± 0.1	82%

**FIGURE 3:**
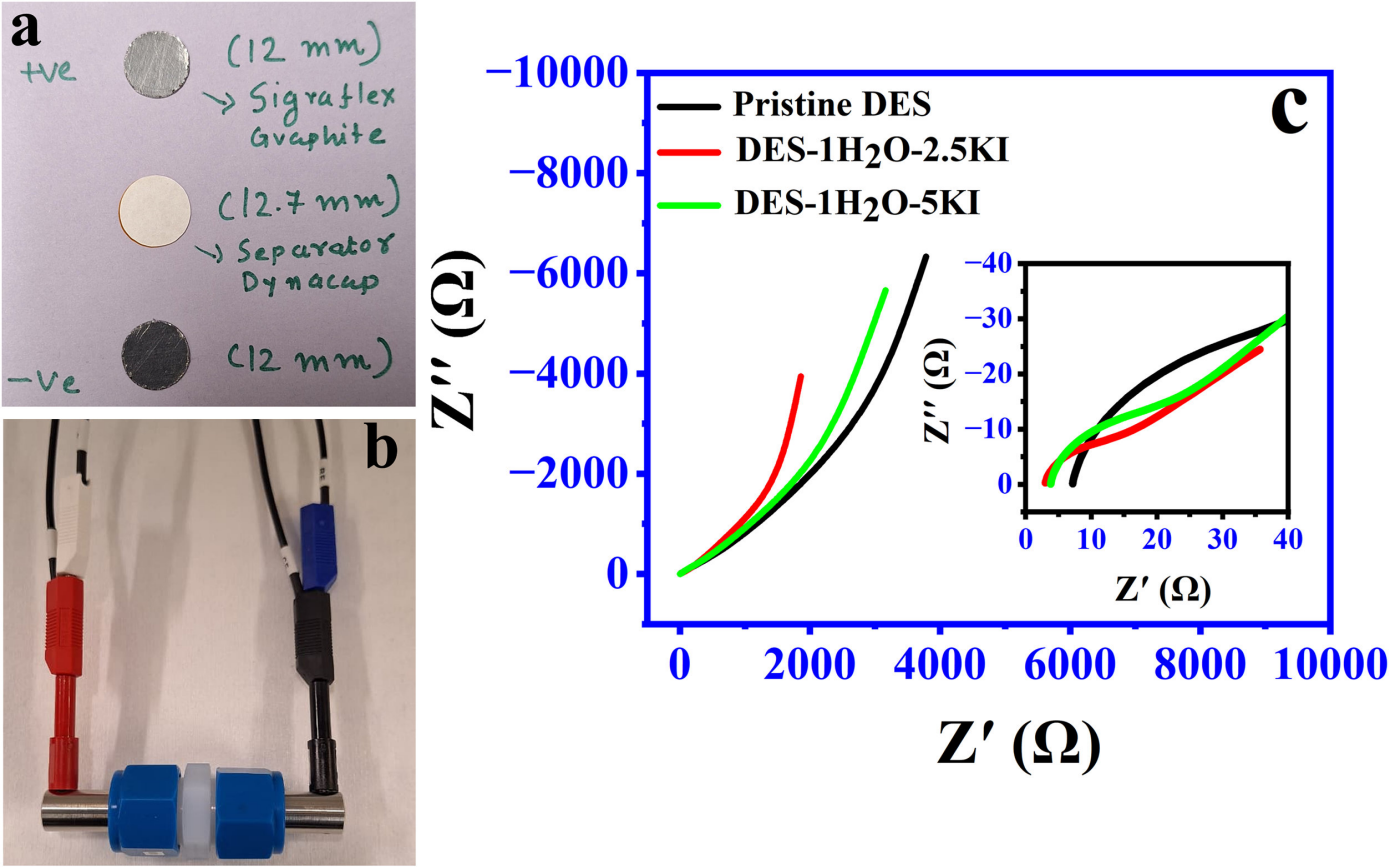
Experimental setup used for ionic conductivity measurements and corresponding impedance spectra (a) components of the conductivity cell including SIGRAFLEX flexible graphite foil blocking electrodes and cellulose separator, (b) assembled Swagelok conductivity cell, and (c) Nyquist plots of pristine DES, DES‐1H_2_O‐2.5KI, and DES‐1H_2_O‐5KI electrolytes recorded for ionic conductivity determination.

(6) $$\text{σ}=\frac{L}{R\times A}$$

where *σ* denotes the ionic conductivity (mS cm^−1^), *L* represents the distance between the two electrodes (40 µm), *A* corresponds to the effective cross‐sectional area available for ion transport (1.13 cm^2^ (based on 12 mm diameter electrode)), and *R* is the bulk resistance obtained from the high‐frequency intercept of the Nyquist plot. The Nyquist plots of the investigated electrolyte systems are shown Figure 3c. As shown in Figure 3c, the pristine DES exhibits the largest high‐frequency intercept, corresponding to the highest bulk resistance (7.14 Ω) and consequently the lowest ionic conductivity (~0.50 mS cm^−1^). Notably, this value is in excellent agreement with the ionic conductivity reported for the choline chloride–urea DES system in the literature, confirming the reliability of the measurement method and electrolyte preparation procedure [5]. Upon the addition of KI, the high‐frequency intercept shifts toward lower Z′ values, indicating a reduction in bulk resistance and enhanced ionic conductivity. The optimized DES‐1H_2_O‐2.5KI electrolyte exhibited an ionic conductivity of 1.19 mS cm^−1^, indicating more than a twofold increase compared with the pristine DES. This improvement can be attributed to the increased concentration of mobile ionic species and enhanced charge transport within the electrolyte. The enlarged high‐frequency region further reveals a depressed semicircle associated with interfacial ion‐transfer processes between the electrolyte and the blocking SIGRAFLEX electrodes. The diameter of the semicircle decreases after KI incorporation, indicating lower interfacial resistance and more efficient ion transport kinetics. Although the higher KI concentration introduces a larger number of charge carriers, the conductivity does not increase further. This behavior can be attributed to stronger ion–ion interactions and the formation of a more structured electrolyte environment at elevated KI concentrations, which restricts ion mobility. Consistent with this observation, the enlarged high‐frequency region of the Nyquist plot shows a slightly larger semicircle for DES‐1H_2_O‐5KI compared to DES‐1H_2_O‐2.5KI, indicating increased interfacial resistance and less efficient ion transport. These results suggest that excessive KI addition can partially hinder ionic transport despite the higher concentration of redox‐active species, highlighting the importance of optimizing KI content to achieve the best balance between conductivity and electrochemical performance. Among the investigated electrolytes, DES‐1H_2_O‐2.5KI exhibits the smallest semicircle diameter, suggesting the most favorable ion transport behavior. These results are consistent with the subsequent CV and GCD measurements, where the DES‐1H_2_O‐2.5KI electrolyte showed a favorable balance between ionic transport and electrochemical performance. In contrast, the DES‐1H_2_O‐5KI electrolyte exhibited an ionic conductivity of 0.93 mS cm^−1^, which is lower than that of the DES‐1H_2_O‐2.5KI electrolyte.

To further compare the electrochemical behavior of the optimized and high‐KI electrolytes, Figure 4c,d shows the CV and GCD responses of DES‐1H_2_O‐2.5KI recorded at different scan rates and current densities. Figure 4e,f shows the corresponding CV and GCD responses for DES‐1H_2_O‐5KI under the same conditions. Overall, the results demonstrate that increasing the KI concentration enhances charge storage, while excessive KI addition introduces diffusion limitations associated with increased electrolyte viscosity. Although DES‐1H_2_O‐5KI exhibits higher capacitance and energy density, DES‐1H_2_O‐2.5KI provides a more balanced combination of charge‐storage performance, ionic transport, and electrolyte stability. The averaged electrochemical performance parameters (mean ± standard deviation, *n* = 6 devices for each KI composition) are summarized in Table 1.

**FIGURE 4:**
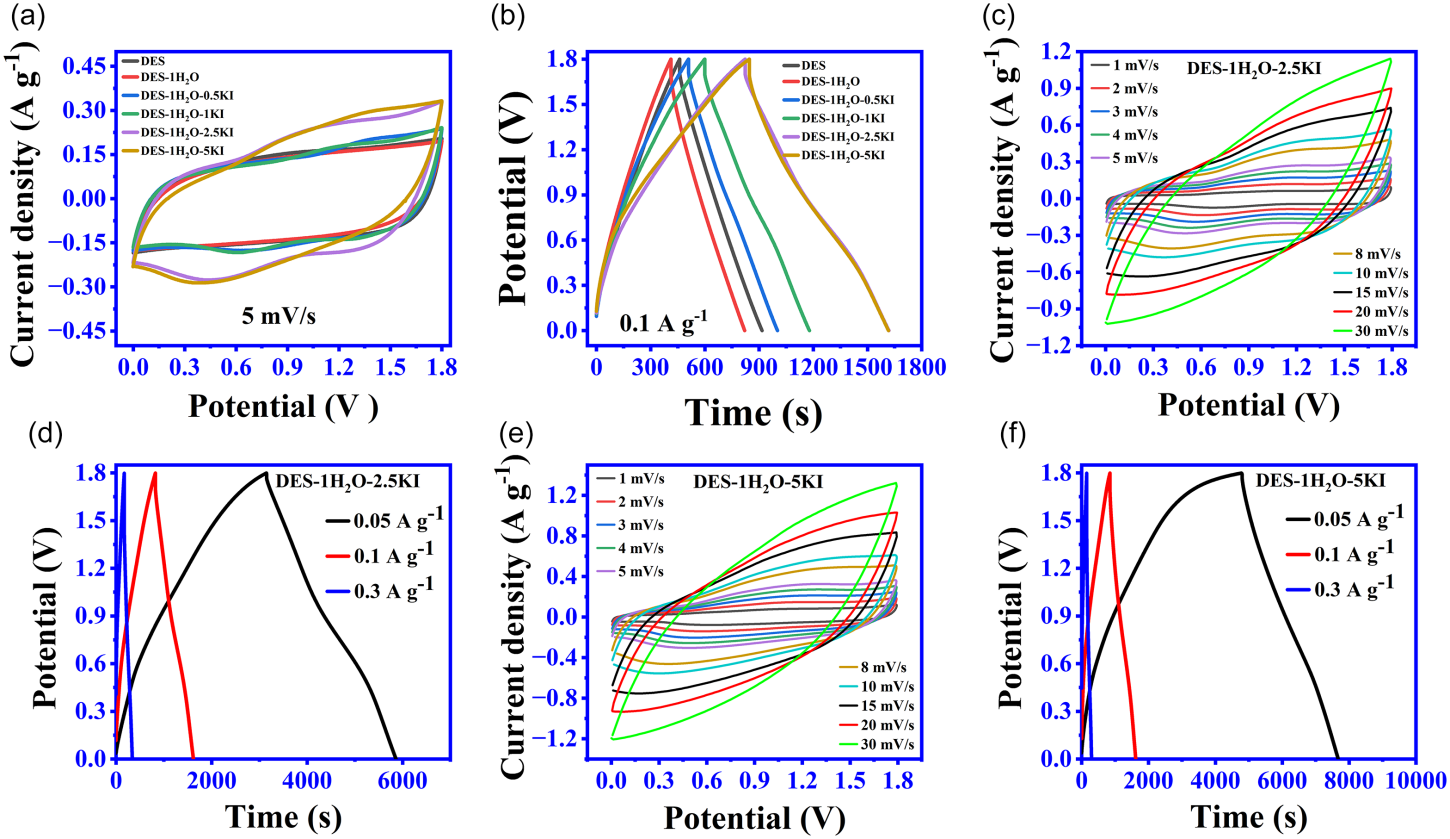
Electrochemical comparison (a) CV curves at 5 mV s^−1^, (b) GCD profiles at 0.1 A g^−1^ of DES‐based electrolytes with varying KI concentrations, (c) CV and (d) GCD curves of DES‐1H_2_O‐2.5KI, (e) CV and (f) GCD performance of DES‐1H_2_O‐5KI.

To gain deeper insight into the charge storage mechanism, the relative contributions of EDLC and Faradaic processes were further investigated using Trasatti analysis for the pristine DES, DES‐1H_2_O‐2.5KI, and DES‐1H_2_O‐5KI electrolytes. The analysis was performed using capacitance values obtained at low scan rates ranging from 1 to 5 mV s^−1^, where diffusion‐controlled redox processes can be more accurately evaluated due to improved ion accessibility within the porous electrode structure. The corresponding results are summarized in Figure 5. Figure 5a–c compares the electrochemical behavior of the investigated electrolytes and clearly demonstrates the effect of KI addition on the charge storage mechanism. The pristine DES electrolytes mainly exhibit EDLC‐type behavior, characterized by relatively stable capacitance values and nearly ideal capacitive response. In contrast, the introduction of KI progressively increases the current response and overall capacitance due to the emergence of additional Faradaic reactions involving the reversible iodide–triiodide redox couple. This indicates that KI functions as a redox‐active electrolyte additive capable of contributing additional charge storage beyond conventional electrostatic adsorption.

**FIGURE 5:**
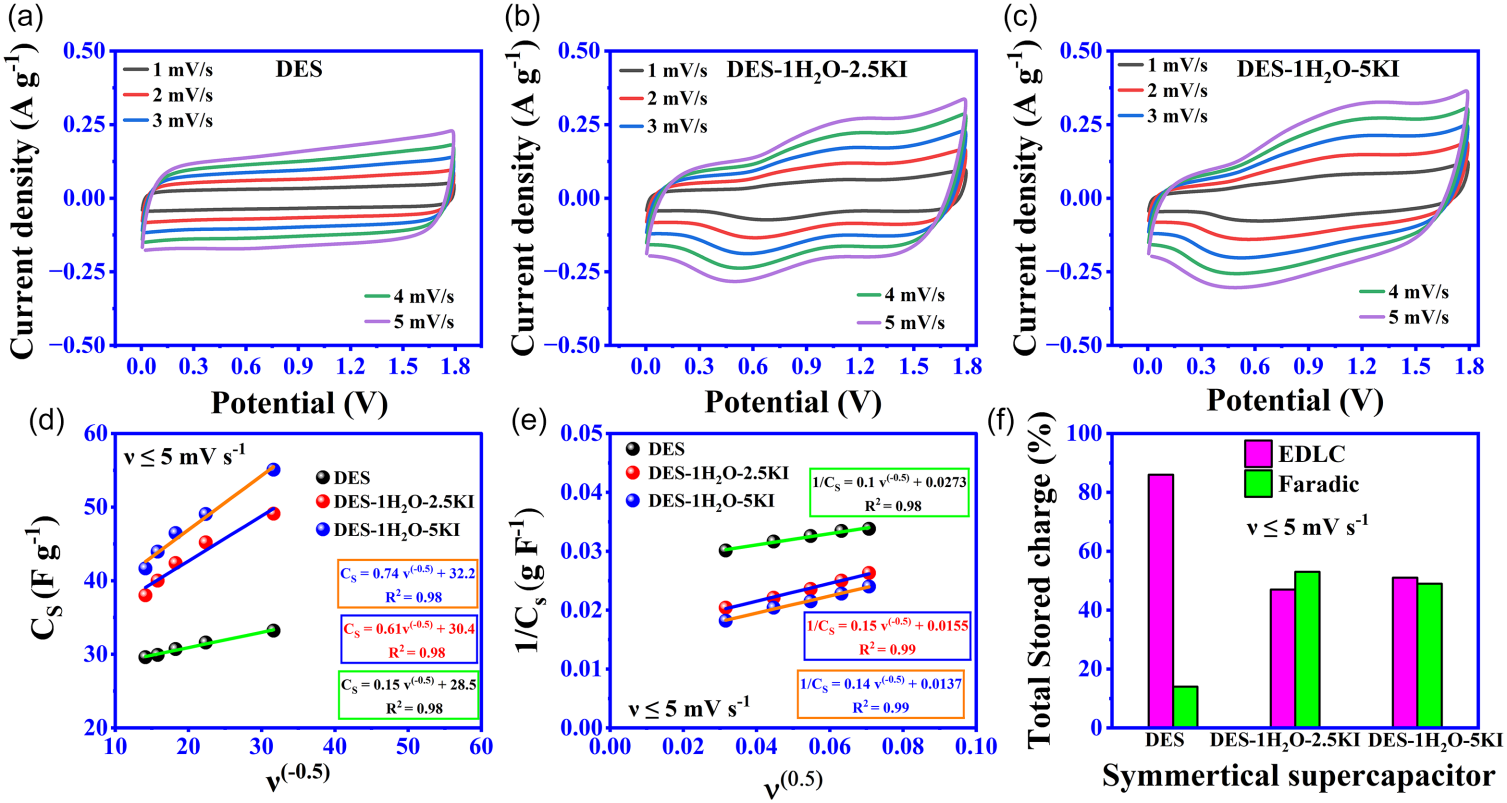
Trasatti analysis of DES‐based electrolytes showing the estimated relative contributions of EDLC and pseudocapacitive charge storage (a–c) CV curves of pristine DES, DES‐1H_2_O‐2.5KI, and DES‐1H_2_O‐5KI recorded at different scan rates, (d) C versus ʋ^(−0.5)^, (e) (1/C) versus ʋ^(0.5)^ plots for all electrolyte system obtained at low scan rates (1–5 mV s^−1^) and (f) estimated relative EDLC and pseudocapacitive contributions derived from Trasatti analysis.

To quantitatively distinguish the EDLC and pseudocapacitive contributions, Trasatti analysis was carried out for the optimized DES‐1H_2_O‐2.5KI electrolyte, as shown in Figure 5d,e. Figure 5d presents the linear relationship between specific capacitance and ʋ^(−0.5)^, where the extrapolated intercept corresponds to the EDLC contribution. Meanwhile, Figure 5e shows the corresponding (1/C) versus ʋ^(0.5)^ relationship, which was used to estimate the total capacitance contribution at infinitely slow scan rates. The good linearity observed in both plots confirms the applicability of the Trasatti model for analyzing the charge storage mechanism of the DES‐based electrolytes. The pristine DES electrolyte exhibited predominantly EDLC‐controlled charge storage, with ≈86% of the total capacitance originating from electric double‐layer formation and only about 14% arising from pseudocapacitive processes. This behavior is consistent with the nearly rectangular CV profiles and triangular GCD curves observed earlier (Figure 5a), confirming that charge storage in the pristine DES mainly occurs through electrostatic ion adsorption at the activated carbon–electrolyte interface. Upon the addition of KI, the pseudocapacitive contribution increased significantly due to the emergence of reversible iodide/triiodide redox reactions.

For the optimized DES‐1H_2_O‐2.5KI electrolyte, the EDLC and pseudocapacitive contributions accounted for ≈47% and 53% of the total capacitance, respectively (Figure 5f). This result indicates that the KI additive effectively introduces additional Faradaic charge storage while still maintaining sufficient capacitive behavior for efficient ion transport and electrochemical reversibility. The balanced contribution between EDLC and pseudocapacitance is also supported by the relatively symmetric CV curves and stable GCD profiles, as shown previously in Figure 4c,d. In the case of the DES‐1H_2_O‐5KI electrolyte, the EDLC and pseudocapacitive contributions were ≈51% and 49%, respectively (Figure 5f), indicating that both charge storage mechanisms contribute almost equally to the overall capacitance. Although the higher KI concentration increases the availability of redox‐active iodide species, the electrochemical response becomes increasingly diffusion‐limited at higher scan rates, as evidenced by the distorted CV profiles and increased polarization observed in Figure 4e,f. This behavior is likely associated with the increased electrolyte viscosity and stronger ion–ion interactions at elevated KI concentrations, which hinder ion mobility within the porous activated carbon structure.

Recent studies of high‐energy hybrid SCs have also reported substantially different relative capacitive and diffusion‐controlled contributions depending on the electrode architecture, electrolyte chemistry, scan rate, and analysis method [21, 23]. These variations indicate that the charge‐storage contributions obtained from kinetic analyses are strongly system‐dependent and should not be directly compared across different SC chemistries. In the present system, the increased contribution beyond conventional EDLC behavior after KI addition supports the presence of additional Faradaic charge storage associated with the redox‐active electrolyte. Overall, the Trasatti analysis suggests that the enhanced charge‐storage behavior of the KI‐containing DES electrolytes arises from the combined contributions of EDLC charge storage and additional Faradaic processes associated with the iodide/triiodide redox couple. Overall, the Trasatti analysis suggests that the improved electrochemical performance of KI‐containing DES electrolytes originates from the combined contributions of EDLC charge storage and additional Faradaic processes associated with the iodide/triiodide redox couple. Among the investigated compositions, DES‐1H_2_O‐2.5KI provides an advantageous balance between capacitive behavior, redox enhancement, ion transport, and electrochemical stability, resulting in enhanced electrochemical performance without significant diffusion limitations.

Figure 6a shows the cycling stability of the devices using different DES‐based electrolytes over 10,000 charge–discharge cycles, and the corresponding capacitance retention values are also summarized in Table 1. The pristine DES and DES‐1H_2_O electrolytes exhibit relatively poor capacitance retention, retaining only around 82%–85% of their initial capacitance after prolonged cycling. The addition of KI significantly improves the cycling performance, particularly for the DES‐1H_2_O‐0.5KI, DES‐1H_2_O‐1KI, and DES‐1H_2_O‐2.5KI electrolytes, which maintain capacitance retention values close to 97%–99% after 10,000 cycles. The improved stability can be attributed to the reversible iodide/triiodide redox reactions, which act as an additional charge storage mechanism while remaining electrochemically reversible during repeated cycling. The stable cycling response indicates that the iodide‐based redox contribution remains largely reversible under the applied cycling conditions. In contrast, the DES‐1H_2_O‐5KI electrolyte exhibits noticeably lower capacitance retention compared to the moderate KI concentrations despite its higher initial capacitance. This behavior likely originates from the excessive concentration of iodide species, which can increase electrolyte viscosity and promote stronger ion interactions within the DES matrix. As a result, ion transport inside the porous activated carbon structure becomes less efficient during prolonged cycling, leading to gradual polarization and reduced electrochemical stability. This observation agrees well with the earlier rheological results and the partial crystallization behavior observed for the 5 wt% KI electrolytes during storage. Figure 6b presents the Nyquist plots of the electrolytes investigated before and after cycling. All samples exhibit a small semicircle in the high‐frequency region followed by a sloped line in the low‐frequency region, which are characteristic features of charge‐transfer resistance and ion diffusion behavior in porous carbon‐based SCs. The relatively small semicircle diameter observed for the KI‐containing electrolytes indicates lower interfacial charge‐transfer resistance compared to the pristine DES system, suggesting improved electrochemical kinetics after KI addition. Furthermore, the Nyquist plots recorded after cycling show only minor shifts for the DES‐1H_2_O‐2.5KI electrolyte, indicating stable electrode/electrolyte interfaces and good reversibility of the iodide‐based redox reactions during long‐term cycling. In comparison, the pristine DES and DES‐1H_2_O electrolytes show slightly larger impedance changes after cycling, reflecting gradual deterioration of ion transport and interfacial conductivity. The DES‐1H_2_O‐5KI electrolyte also exhibits a larger impedance response after cycling compared to the optimized 2.5 wt% KI system, supporting the conclusion that excessive KI concentration introduces diffusion limitations and less stable ion transport behavior during repeated operation.

**FIGURE 6:**
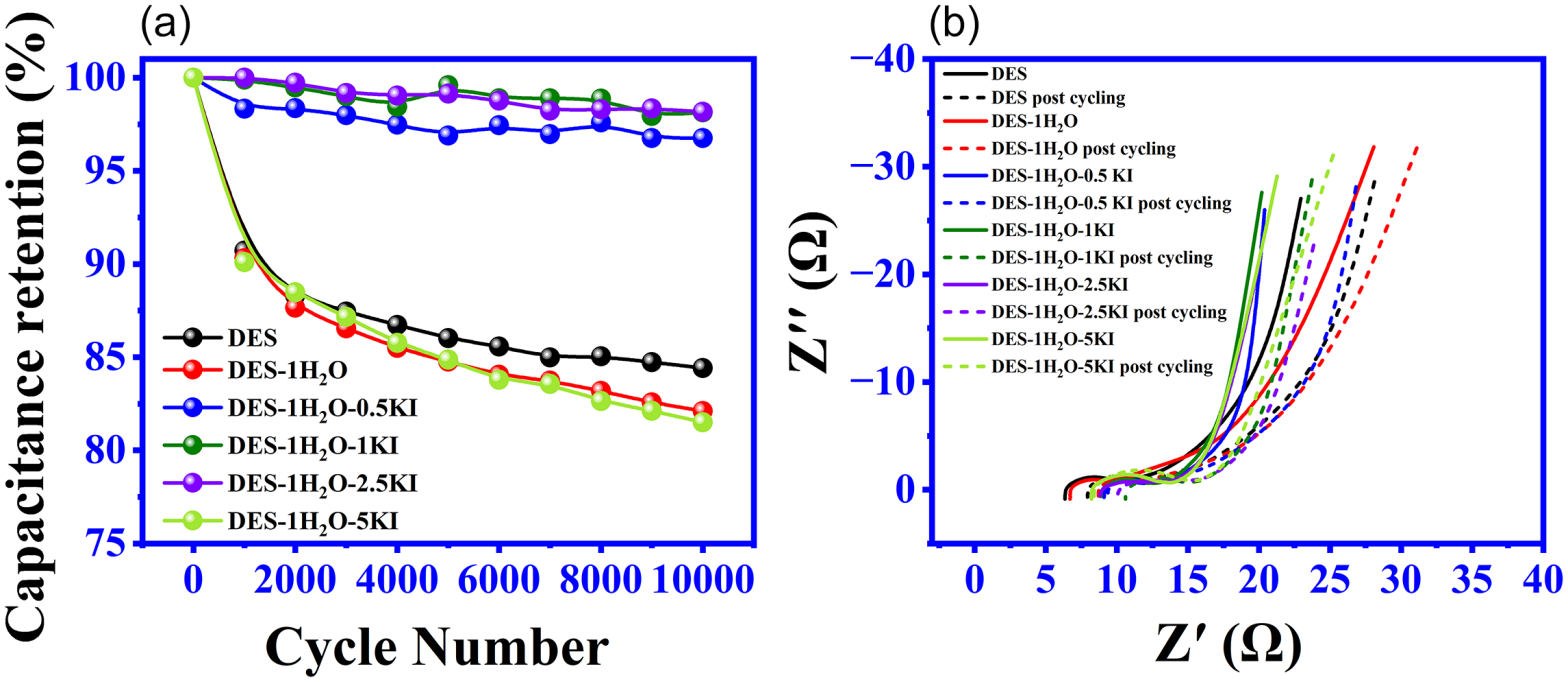
(a) Capacitance retention of DES‐based SCs during 10,000 charge–discharge cycles showing the influence of KI concentration on long‐term electrochemical stability and (b) Nyquist plots of DES, DES‐1H_2_O, DES‐1H_2_O‐0.5KI, DES‐1H_2_O‐1KI, DES‐1H_2_O‐2.5KI, and DES‐1H_2_O‐5KI electrolytes before and after cycling.

To better understand the enhanced electrochemical behavior of the KI‐containing DES electrolytes, the charge storage mechanism of the symmetric SC system was further examined. In the pristine DES electrolyte, charge storage mainly occurs through EDLC, where electrolyte ions are electrostatically adsorbed on the porous activated carbon surface during charging and released during discharging. In this process, choline cations preferentially migrate toward the negatively polarized electrode, while chloride ions (Cl^−^) accumulate near the positive electrode. After introducing KI into the DES‐1H_2_O electrolyte, additional Faradaic charge storage is attributed to reversible iodide‐based redox processes. During charging, iodide ions near the positively polarized electrode are proposed to undergo oxidation with the formation of triiodide species according to the reversible reaction [10, 13].

$$3I^{\;\text{−}}\Leftrightarrow I_{3}^{-}+2e^{-}$$

This proposed reaction provides an additional Faradaic charge‐storage pathway alongside conventional electrostatic ion adsorption at the porous activated carbon–electrolyte interface. Within the porous activated carbon electrode, the redox‐active iodide species can access electrolyte‐filled pores and contribute to Faradaic charge storage in addition to electrostatic ion adsorption at the carbon/electrolyte interface. At higher KI concentrations, increased viscosity and ion–ion interactions can restrict transport into these pores, contributing to the observed diffusion limitations. The chloride ions originating from choline chloride are considered part of the hydrogen‐bonded DES structure and are not proposed to participate directly in the redox reaction. Instead, the choline chloride–urea matrix provides the electrolyte environment in which the iodide‐based redox species are transported and accessed within the porous carbon electrode. The corresponding charge storage mechanism for the DES‐1H_2_O‐KI electrolyte is schematically illustrated in Figure 7. The proposed reversible iodide/triiodide conversion is consistent with additional Faradaic charge storage alongside the conventional EDLC behavior of activated carbon. At the same time, potassium ions (K^+^) contribute to ionic charge compensation and transport within the electrolyte during ion migration.

**FIGURE 7:**
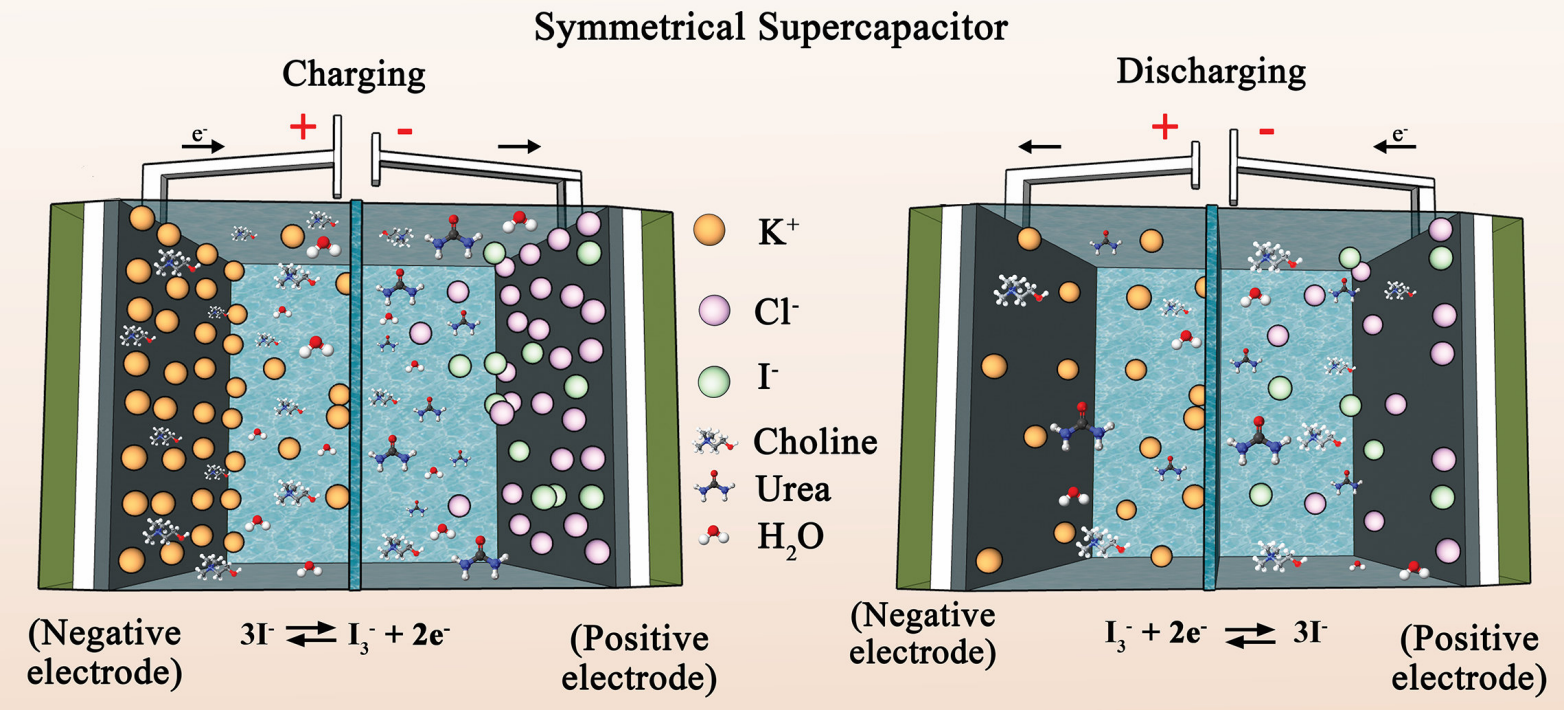
Charge storage mechanism for the DES‐1H_2_O‐KI electrolyte.

During discharge, the reverse reduction process is proposed to reduce the oxidized iodine species back toward iodide, releasing the stored Faradaic charge. This reversible process occurs alongside ion desorption from the activated carbon surface during EDLC discharge. The coexistence of electrostatic ion adsorption and reversible iodide redox reactions explains the improved capacitance observed for the DES‐1H_2_O‐KI systems, particularly for the DES‐1H_2_O‐2.5KI electrolyte. The enhanced pseudocapacitive contribution identified from the Trasatti analysis, together with the reduced charge‐transfer resistance observed in the Nyquist plots, confirms that the KI additive improves both charge storage activity and ion transport kinetics within the electrolyte system. The electrochemical performance of the optimized DES‐1H_2_O‐2.5KI symmetric SC was further benchmarked against previously reported activated carbon‐based symmetric SCs, as summarized in Table 2.

**TABLE 2:** Comparison of the electrochemical performance of activated carbon‐based printed symmetric SCs reported in previous studies.

Cell configuration (positive electrode//electrolyte//negative electrode)	Voltage‍ window, V	**Energy ‍density, ‍W ‍h kg** ^**1**^	**Power ‍density, ‍kW kg** ^**−1**^	Capacitance retention rate % (no. of cycles)	Ref.
AC//DES‐1H_2_O‐2.5KI//AC (ChCl–urea+1% H_2_O+KI)	1.8	25	1.2	98% (10,000)	This work
AC//DES//AC (ChCl–urea+1% H_2_O)	2.2	7	1	90% (25,000)	[4]
AC//DES//AC (ChCl–urea)	2	20.6	0.56	90.2% (10,000)	[6]
AC//DES//AC (ChCl–urea)	2	22	1.835	87% (10,000)	[7]
AC//DES//AC DES (KI + EG)	1.4	18.42	0.7	89.8% (3000)	[13]
AC//DES//AC DES (ChCl‐urea)	1.8	14.8	6.7	85% (10,000)	[17]
AC//DES//AC DES(ChCl‐urea)	1.8	22	0.4	90% (10,000)	[16]
AC//DES//AC DES (ChCl–urea)	1.8	7.6	1.6	84% (10,000)	[15]
AC//DES//AC DES (ChCl–urea)	1.8	7.2	1.5	67% (10,000)	[14]
Aqueous electrolyte + KI					
Ti_3_C_2_T_*x*_//H_2_SO_4 _+ KI//Ti_3_C_2_T_*x*_	1	23.19	0.5	100% (5000)	[24]
AC//H_2_SO_4 _+ KI//AC	1	19.04	0.224	100% (4000)	[25]
AC//Na_2_SO_4 _+ KI//AC	1	12.3	—	—	
AC//KOH + KI//AC	1.6	7.1	6.222	93% (14,000)	[26]
AC//PVA‐KOH + KI//AC	1	7.8	15.34	95.76% (10,000)	[27]

Abbreviations: ChCl, choline chloride; EG, ethylene glycol; H_2_SO_4_, sulfuric acid; KOH, potassium hydroxide; Na_2_SO_4_, sodium sulfate; PVA, polyvinyl alcohol; and Ti_3_C_2_T_*x*_, titanium carbide (MXene film).

A comparison with our previously reported work and other activated carbon‐based printed symmetric SCs demonstrates that the optimized DES‐1H_2_O‐2.5KI electrolyte delivers competitive electrochemical performance while maintaining good cycling stability and a low‐cost electrolyte composition. While redox‐active additives can substantially improve the charge storage capability of SCs, their practical application often remains limited by issues such as electrolyte stability, side reactions, self‐discharge, and long‐term cycling performance. In this regard, the present study demonstrates that incorporating a controlled amount of KI into a DES electrolyte can enhance charge storage while maintaining excellent electrochemical stability, highlighting a promising strategy for the development of sustainable redox‐enhanced SCs. Future studies will focus on exploring alternative sustainable redox‐active additives and evaluating the biodegradability and environmental impact of DES‐based electrolyte systems to further improve the performance and sustainability of printed SCs.

## 4 Conclusions

The electrochemical results demonstrate that the incorporation of KI significantly influences the charge storage behavior of the DES‐based electrolyte system. Among the investigated compositions, the DES‐1H_2_O‐2.5KI electrolyte provided the most balanced combination of charge storage, ionic transport, and cycling stability, although the DES‐1H_2_O‐5KI electrolyte exhibited higher specific capacitance and energy density. Trasatti analysis further suggested that the improved performance originates from the synergistic contribution of EDLC behavior and reversible iodide‐mediated pseudocapacitive reactions. In contrast, excessive KI concentration led to reduced electrochemical efficiency, likely due to increased ion crowding and diffusion limitations within the electrolyte. These findings highlight the importance of electrolyte optimization in regulating ion transport and redox activity and demonstrate the strong potential of DES‐KI systems as low‐cost and environmentally friendly electrolytes for next‐generation sustainable SC applications.

## Author Contributions

**Chirag Mevada**: conceptualization, methodology, validation, formal analysis, investigation, data curation, writing – original draft, visualization, and project administration. **Aapo Kattainen:** investigation, data curation, writing – original draft. **Jari Keskinen:** validation, writing ‍– original draft, resources, supervision. **Matti Mäntysalo:** validation, resources, writing – review & editing supervision, project administration, and funding acquisition.

## Funding

Funded by the European Union. This publication was supported by the SUINK project funded by the European Union’s Horizon Europe Research and Innovation Program under Grant Agreement No. 101070112.

## Conflicts of Interest

The authors have no financial or personal relationships that could influence the work presented in this article.

## Data Availability

The data that support the findings of this study are available on request from the corresponding author. The data are not publicly available due to privacy or ethical restrictions.
